# Parliament and the Profession

**Published:** 1916-03-18

**Authors:** 


### PARLIAMENT AND THE PROFESSION.

### The New Hampshire Mental Hospital.

Replying to Mr. King, Mr. Brace stated that the new Hampshire Mental Hospital is to be used by the military authorities as a hospital for sick and wounded soldiers, and not for cases of mental and nervous affections. No other new mental hospital is now in actual progress, the building of two new ones having been suspended; and one or two contemplated schemes are held in abeyance.

### Napsbury Mental Hospital.

The whole of the Middlesex County Mental Hospital at Napsbury is being handed over to the War Office for use as a military hospital, and the patients there are being distributed over other mental hospitals as near as practicable to Middlesex. The hospital is to be used by the War Office for sick and wounded soldiers, but, explained Mr. Brace in reply to Mr. King, it is still intended to receive there a certain number of soldiers suffering from mental and nervous affections.

### Invalid Prisoners in Switzerland.

The fact alluded to in The Hospital last week (p. 534) that certain French prisoners of war are to be treated in Swiss hospitals has been mentioned three times in the House of Commons. In reply to Mr. Malcolm, the Prime Minister said that action was being taken by the Government to secure that a number of British prisoners suffering from specified diseases should be treated in hospitals in Switzerland, but subsequently, in reply to another question, Mr. Tennant said he was not in a position to say how soon the first British invalid prisoners would be sent to Swiss sanatoria. We learn that the French Government is paying for the treatment and accommodation at the rate of 5 francs a day for a private and 8 francs for an officer.

### Red Cross Visitors to British War Prisoners.

During the present winter three Russian Red Cross ladies, accompanied by three neutrals, have visited over seventy prisoner camps in Germany and a large number of camps in Russia, but, replying to Mr. Malcolm, Lord Robert Cecil stated that His Majesty's Government could not consent to a similar interchange of visits between Red Cross representatives of Germany and England accompanied by neutrals. He added that the members of the staff of the United States Embassy at Berlin had been good enough to devote a considerable proportion of their time to the care of British prisoners.

### Medical Examiners' Fees.

The existence of curious ideas with regard to the terms on which the medical examination of recruits is conducted has been revealed by several questions addressed to Ministers. Thus Mr. Snowden asked if doctors were paid only for recruits whom they pass as sound, and whether this could be accepted as the explanation of "the fact that recently men have been passed as fit for military service who have an artificial leg, men who are so mentally deficient that they are unable to understand or answer any question put to them by the recruiting officer, and men who have never done any work for years on account of daily attacks of epilepsy." In his reply, Mr. Tennant said he welcomed the opportunity for contradicting categorically the statement that recruiting medical officers are paid only for recruits whom they pass as sound. "It is," he said, "the examination which is paid for, and the pecuniary result to the examining medical officer is the same whether he accepts or rejects a recruit." He referred to the cases mentioned by Mr. Snowden as an incorrect representation of the results of the work of the examining medical officers. "I think," he added, "that instead of being held up to obloquy they deserve support and recognition in the heavy work they are patriotically doing on behalf of the country." Answering other questions, Mr. Tennant said that, subject to certain limitations, the usual fee for medical examination is 2s. 6d. per man, and that if within three months the man was found to have been improperly passed, the doctor was liable to refund the fee.

### Surgical Operations at the Front.

Mr. Tennant described as "a figment of some fertile imagination" the suggestion that at the outbreak of war an order was in force forbidding medical officers of the Royal Army Medical Corps to operate in abdominal cases. He added that in many instances a man in need of a grave operation had found himself on the operating table within three or four hours of his being shot, and that there were cases in which operations upon the skull and the abdomen had been carried out within two hours and a half of the receipt of the wound. There is an organisation of clearing stations so near the Front that they are constantly under shell fire, in which these operations can and do take place at all hours of the day and night.

### Lunacy Commissioners and the War.

In consequence of the taking over by the War Office of a number of mental hospitals, Mr. King suggested that the work of the Lunacy Commissioners had been largely curtailed, and that therefore a useful economy might be effected by reducing the salaries of the Commissioners. Mr. Herbert Samuel disposed of this suggestion by pointing out that two of the Commissioners had undertaken the continuous supervision of the War Hospitals scheme, while another Commissioner had been absent on military duty since the beginning of the war, and more than half the clerical staff had joined the Forces. On the other hand, the concentration of civil patients into a smaller number of mental hospitals had demanded even closer supervision than formerly. The expenditure of the Board, however, has been reduced in many directions. and the estimates for the coming year will show a large reduction.

### Other Matters of Interest.

Cerebro-spinal Meningitis in the Portsmouth Area.—In the Portsmouth district medical administrative area thirty-one cases of cerebro-spinal meningitis have occurred among soldiers, and the number of deaths has been eight. In giving this information, in reply to a question, Mr. Tennant added that the special measures taken to guard against the spread of the disease consisted of isolation of the patient, disinfection of the patient's clothing, etc., segregation and special medical treatment of contacts and carriers.

Non-poisonous Varnish for Aeroplanes.—The main difficulty in the way of the adoption of a non-poisonous dope for aeroplane wings is that one of the essential ingredients is not produced commercially in this country at present. In reply to Mr. Rowlands, however, Mr. Brace stated that efforts were being made to increase the supply of this ingredient.

